# Integrated Proteomic and Transcriptomic Analysis Reveals the Mechanism of Selenium-Mediated Cell Wall Polysaccharide in Rice (*Oryza sativa* L.) Cadmium Detoxification

**DOI:** 10.3390/toxics13080642

**Published:** 2025-07-30

**Authors:** Sixi Zhu, Xianwang Du, Wei Zhao, Xiuqin Yang, Luying Sheng, Huan Mao, Suxia Su

**Affiliations:** The Karst Environmental Geological Hazard Prevention of Key Laboratory of State Ethnic Affairs Commission, College of Eco-Environment Engineering, Guizhou Minzu University, Guiyang 550025, China; 18103516573@163.com (X.D.); 17312887996@163.com (W.Z.); yangxiuqing0928@163.com (X.Y.); sly18504232925@163.com (L.S.); maohuan0710@163.com (H.M.); 17851141801@163.com (S.S.)

**Keywords:** cadmium contamination, proteomics, transcriptomics, mult-omics, cell wall

## Abstract

Cadmium (Cd) toxicity destroys plant cells and affects plant growth and development. Due to its unique metallic properties, selenium (Se) has been shown to be effective in antioxidants, cellular immunity, and heavy metal detoxification. When Se and Cd are present together in plants, they antagonize. However, the mechanism of action of the two in the rice cell wall remains to be clarified. In this study, we analyzed the mechanism of Cd detoxification by rice (*Oryza sativa* L.) cellular polysaccharides mediated by Se, using the cell wall as an entry point. Proteomic and transcriptomic analyses revealed that “Glycosyl hydrolases family 17”, “O-methyltransferase”, and “Polygalacturonase” protein pathways were significantly expressed in the cell wall. The most abundant enzymes involved in polysaccharide biosynthesis were found, including bglB, otsB, HK, PFP, ADH1, and ALDH, which resulted in the synthetic pathway of polysaccharide formation in the rice cell wall. Finally, the essential genes/proteins, such as protein Os03g0170500, were identified. The study showed that Se inhibits Cd uptake and transport when Se (1 mg/kg) is low relative to Cd (3 mg/kg), has little inhibitory effect, and even promotes Cd (3 mg/kg) uptake when Se (5 mg/kg) is relatively high.

## 1. Introduction

Human civilization’s progress is often inseparable from industrialization’s rapid development, and pollution comes with it. Heavy metal pollution has now seriously affected agricultural production [1]. Soil cadmium (Cd) contamination poses a major threat to global food security and human health [2]. China is the largest Cd metal-producing area, topping the list with a 41.67% share in 2021. Cd is a typical toxic heavy metal element, which not only breaks the normal physiological metabolism of plants but also enters the human body through the food chain and impacts human health. [3]. With the aggravation of heavy metal pollution, China’s usable arable land area has been drastically reduced, of which the Cd-contaminated arable land area is as high as 2.786 × 10^3^ km^2^ [4]. Selenium (Se) has the unique property of scavenging free radicals in plants and has significant effects on antioxidants, cellular immunity, and heavy metal detoxification [5,6]. However, due to its narrow safety threshold, excessive amounts of Se can also harm the environment [7]. Animal studies have shown antagonism between Se and Cd [8]. Physiological mitigation [9], uptake and translocation inhibition, and their potential mechanisms [10] between Se and Cd have been investigated in the plant field. In summary, studying the role of plant cell walls in Cd detoxification mediated by Se can effectively solve the problem of Cd contamination in crops.

The roots of plants are the primary organ for absorbing nutrients from the soil and the main organ for absorbing and transferring Cd [11,12]. Excessive Cd^2+^ accumulation can disrupt the plant photosynthetic system, inhibit photosynthetic pigment synthesis, interfere with C, N, and P nutrient metabolic pathways, induce free radical production, exacerbate the degree of membrane lipid peroxidation, and lead to the abnormal expression of functional proteins [13,14]. Therefore, plants have evolved their defense mechanisms to cope with Cd toxicity under long-term Cd stress. It has been shown that in the process of heavy metal ions entering the plant, they first have to break through the blockage of the root cell wall, and in this process, a part of Cd^2+^ will remain in the cell wall [15]. Cellulose (hemicellulose-1 and hemicellulose-2) is the main constituent of the cell wall, along with a large number of matrix polysaccharides (hemicellulosic polysaccharides (glucans, heteromannans) and pectic polysaccharides) and lignin [16]. Functional groups (-NH_2_, -COOH, -SH, and -OH) can mitigate the toxic damage caused by heavy metal ions by binding to them at the cellular molecular structure level [17,18]. At the same time, pectin also contains many negatively charged groups that are easy to combine with heavy metal ions, which significantly enhances the ability of the cell wall to fix metal ions [19,20]. Moreover, metal ions can bind to hemicellulose to form metal–hemicellulose complexes and alleviate their toxicity [21].

In response to environmental heavy metal stress, plants can reduce cellular damage by regulating their polysaccharide biosynthesis pathways and enhancing cell wall biological functions to bind more heavy metal ions and limit their translocation processes in the plant [22]. Among them, the polysaccharide biosynthesis pathway is divided into three main processes. First, in plants, β-furanofructosidase (INV) acts on sucrose to convert it to glucose 6-phosphate (Glc-6P) and fructose. The resulting fructose is then catalyzed by hexokinase (HK) and fructokinase to produce fructose 6-phosphate (Fru-6P) [18]. Next, Glc-6P to glucose 1-phosphate (Glc-1P) was catalyzed by phosphoglucomutase (pgm), and the remaining Glc-6P and Glc-1P precursor substances were generated into guanosine diphosphate mannose (GDP-Man) and UDP-glucose (UDP-Glc), respectively. Second, GDP-Man and UDP-Glc generate several other NDP sugars in the presence of nucleotide-diphosphate-sugar (NDP-sugar) interconverting enzymes (NSEs) [15]. Ultimately, NDP-sugar generates polysaccharides in response to glycosyltransferase (GT) reactions [20].

Rice (*Oryza sativa* L.) is one of the five cereals and an indispensable food crop in China [13]. More than 90% of rice is produced in Asian countries, and the area under rice cultivation in China in 2024 will be about 29,006 hectares. Cultivated land is being eroded by heavy metal pollution, and Cd contamination may threaten food production safety. The mechanisms of cell wall remodeling in plants under Se and Cd stress are still poorly understood. Particularly concerning the cell wall polysaccharide biosynthesis pathway, which involves changes in functional genes and proteins, has yet to be fully revealed. In this study, we integrated physiological, transcriptomic, and proteomic approaches to investigate the effects of Se-mediated Cd stress on the candidate genes, functional proteins, and pathways of cell wall polysaccharide biosynthesis in rice roots. We revealed the regulatory mechanism of cell wall polysaccharides. This finding will contribute to a better understanding of the molecular response mechanism of the rice root system to Cd stress and provide new ideas for inhibiting heavy metal transport to edible organs of crops to achieve food production security. Therefore, how to decrease Cd in rice by Se has become a hot topic of current research.

## 2. Materials and Methods

### 2.1. Plants and Growing Conditions

**Figure 1:**
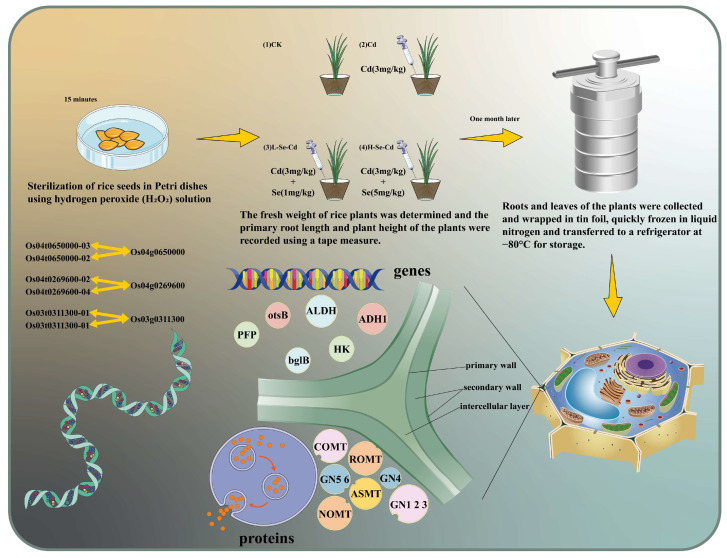
Experimental flow chart.

The rice variety used in this experiment was Chunyou 987 Japonica. This variety is a Japonica-type three-line hybrid rice bred by the China Rice Research Institute, which has been planted on a large scale in China. To avoid the influence of the external environment on the experiment, we chose to cultivate rice in a room with a relative humidity of 70% ± 5%, a constant temperature of 25 °C, a light–dark cycle of 14/10 h, and a light intensity of 176 μmol m^2^/s. We used 1/4 Hoagland nutrient solution (Table S1) for rice cultivation and watered every 5 days to promote germination and growth. Rice seeds of similar size with intact kernels were first sterilized by soaking in H_2_O_2_ and then repeatedly rinsed well. The seeds were then placed in Petri dishes at a constant temperature of 25 °C and kept in the dark for 2 days, alternating with 5 days of light to promote germination. After germination, rice seedlings were transferred to pots (with 350 g of soil in the pots), after which 150 g of soil was evenly covered; the soil used in this experiment was sterilized rice soil with a pH of 6.6. Three pots were planted for each treatment, with about 30 seedlings per pot. In this experiment, the concentrations of Se and Cd were selected according to the relevant standards (GB15618-2018) [23], with a Cd concentration of 3 mg/kg, a low Se concentration of 1 mg/kg and a high Se concentration of 5 mg/kg. Cd and Se were added to the soil as CdCl_2_ and Na_2_SeO_3_ solutions, respectively. Therefore, four treatments were designed in this experiment, namely, CK group without Cd and Se addition, Cd group with 3 mg/kg Cd addition, L-Se-Cd group with 3 mg/kg Cd and 1 mg/kg Se addition, and H-Se-Cd group with 3 mg/kg Cd and 5 mg/kg Se addition, where L-Se-Cd group and H-Se-Cd group are abbreviated as L and H, respectively. The experimental flow is shown in Figure 1.

### 2.2. Measurement and Sampling of Growth Parameters

Photographs were taken every 5 days to record the growth of rice seedlings, and plant samples were collected after 15 days for experimental determination. Firstly, most of the soil in the pots was removed in a sterile environment, after which the soil fixed to the roots of the plants was carefully collected, and the roots of the plants were repeatedly rinsed to obtain complete and clean plants. The collected rhizosphere soil was dried naturally, sieved, and then stored in sealed bags for subsequent determination of soil physicochemical properties and heavy metal content [24]. In each treatment, three rice seedlings were selected and used to determine the plant’s fresh weight, height, and primary root length (Figure S1). Finally, the roots and leaves of the plant samples were separated, wrapped separately in tin foil, and transferred to a refrigerator for storage after rapid freezing in liquid nitrogen for subsequent determination of enzymatic and non-enzymatic antioxidant indices, oxidative stress, and proteomic and transcriptomic analyses.

### 2.3. Cadmium Content Analysis 

In this experiment, Cd was measured in soil samples as well as in plant samples. A total of 0.5 g of each soil sample was added to the ablution tube, followed by aqua regia (1:3 ratio of concentrated hydrochloric acid to concentrated nitric acid), placed in a fume hood, and heated to ablution using an electric hot plate. It was first digested at 160 °C for 1 h. After warming up to 200 °C, 3 mL of HClO_4_ was added and digested for another 1 h. The liquid was then dissolved in a volumetric flask with 1% dilute nitric acid. After completion of the digestion, the liquid was transferred to a volumetric flask and fixed with 1% dilute nitric acid in order to measure the Cd content in the soil samples. The leaf samples were dried in an oven and then ground. 0.5 g of the milled leaf sample was added with 37% HCl and 63% HNO_3_, sealed, and disintegrated in an oven. Finally, the suspension was diluted with 3 mL of HNO_3_, and the Cd content of the leaf samples was measured [25,26]. ICP-MS (Agilent, 7800 ICP-MS, Santa Clara, CA, USA) was used to analyze the Cd content of the samples in this experiment.

### 2.4. Cadmium Enrichment Factors and Transporters 

The enrichment factor (EF) is the ratio of the total Cd content in rice plants (roots and leaves) to the total Cd content in the soil, and the transport factor (TF) is the ratio of the Cd content in rice leaves to the Cd content in rice roots [24].

### 2.5. Plant Physiological and Biochemical Indicators 

We examined the stems of collected rice seedlings for physiological and biochemical indices, including citrate, succinate dehydrogenase (SDH), tyrosine ammonia lyase (TAL), and protopectin and hemicellulose contents. The detection principle of citrate is that under acidic conditions, by determining the increase in absorbance value at 545 nm, the content of citrate in rice samples can be calculated. Citrate content was measured using a citrate content test kit (Comin Biotechnology Co., Ltd., Suzhou, China). SDH catalyzes the dehydrogenation of succinic acid to produce fumarate and the reduction of 2,6—dichlorophenol indophenol (DCPIP) via the hydrogen transfer of phenazine methosulfate (PMS). The rate of reduction of 2,6-DCPIP was determined by the change in absorbance at 600 nm, which can then be used to characterize the activity of SDH. Measurements were performed using an SDH test kit (Comin Biotechnology Co., Ltd., Suzhou, China). TAL can decompose tyrosine to produce coumaric acid so that the absorbance of the reaction solution at 333 nm rises with the reaction time. TAL’s activity can be calculated from the rate of change of absorbance. Measurements were performed using a TAL test kit (Comin Biotechnology Co., Ltd., Suzhou, China). Protopectin is enzymatically hydrolyzed to soluble pectin in dilute acid, after which it is converted to galacturonic acid. The product condenses with carbazole in strong acids to yield a purplish-red compound with a characteristic absorption peak at 530 nm, and the change of absorbance could determine the content of protopectin in rice samples. Measurements were made using a protopectin content test kit (Comin Biotechnology Co., Ltd., Suzhou, China). In contrast, hemicellulose is converted to reducing sugar after acid treatment, which produces a reddish-brown substance with 3,5-Dinitrosalicylic acid (DNS), with a characteristic absorption peak at 540 nm and a considerable absorbance value reflecting the hemicellulose content. Measurements were made using a hemicellulose content test kit (Comin Biotechnology Co., Ltd., Suzhou, China).

### 2.6. Proteome Analysis 

Protein extraction is done by grinding and suspending the plant leaves, followed by centrifugation, before the protein can be extracted from the precipitate. A filter-assisted sample preparation procedure can digest the proteins, and their peptide content can be estimated. Most of the proteins in the data can be found in the UniProt-GOA database, which was annotated by the GO database for proteome categorization and the KEGG database for protein pathway annotation. Protein expression differences were analyzed using a t-test. To ensure the reliability of the data, a two-tailed Fisher’s exact test was also performed to examine GO and KEGG pathways and domain enrichment for differentially expressed proteins. Finally, data were also corrected for multiple hypothesis testing using standard FDR controls, and *p* ≤ 0.05 after the correction was considered significant.

### 2.7. Transcriptome Analysis 

The first step was RNA extraction. Total RNA in the samples was extracted with TRIzol^®^ reagent, and then the quality of the RNA was determined and quantified using the Agilent 5300 fragment analyzer and ND-2000 (NanoDrop Technologies, Waltham, MA, USA), respectively. Shanghai Majorbio Bio-pharm Biotechnology Co. (Shanghai, China) carried out the transcriptome part of this experiment, which included RNA purification, reverse transcription, library construction, and sequencing. Raw reads were processed by fastp (https://github.com/OpenGene/fastp, 6 June 2023) to obtain a clean data image. The obtained clean data were then mapped to the reference genome sequence of rice. When the genome sequences matched exactly, or there was only one mismatch, reads could be annotated based on the reference genome for the following analysis step. The data from this experiment were mainly used to annotate gene functions by Swiss-Prot, NR, KOG/eggNOG, Pfam, KEGG, and GO databases. The quantification of gene expression levels was estimated in this experiment using the fragments per kilobase per million transcript fragments (FPKM) mapping method. Differential expression between samples was analyzed using the EBSeq R software package (version 4.3.2). During the analysis, FDR < 0.05 and |log2 FC (fold change)| ≥ 1 were defined as thresholds for significant differential expression. FDR (False Discovery Rate) is inevitably generated in the data analysis process, and PPDE (Posterior Probability of DE) is chosen as the method to adjust it in this experiment. GO and KEGG enrichment analyses were performed for all DEGs to obtain a more detailed description [27].

### 2.8. Statistical Analysis 

The data from this experiment are the mean ± standard deviation of three replications. The Shapiro–Wilk test and Levene’s test were used to verify the normality and homogeneity of the experimental data. Any treatment differences were analyzed using two-way ANOVA followed by Tukey’s HSD post-processing. Outcome data that did not meet the assumptions of normal distribution and homogeneity of variance were analyzed again with the nonparametric Kruskal–Wallis test. Statistical analyses were performed using IBM SPSS 27.0, setting *p* ≤ 0.05 as the significance level. Visual representations of proteomic and transcriptomic data, such as Venn diagrams, heat maps, and volcano maps, were performed through an online platform (www.majorbio.com, 6 June 2024).

## 3. Results

### 3.1. Indicators of Rice Physiology and Biochemistry

**Figure 2:**
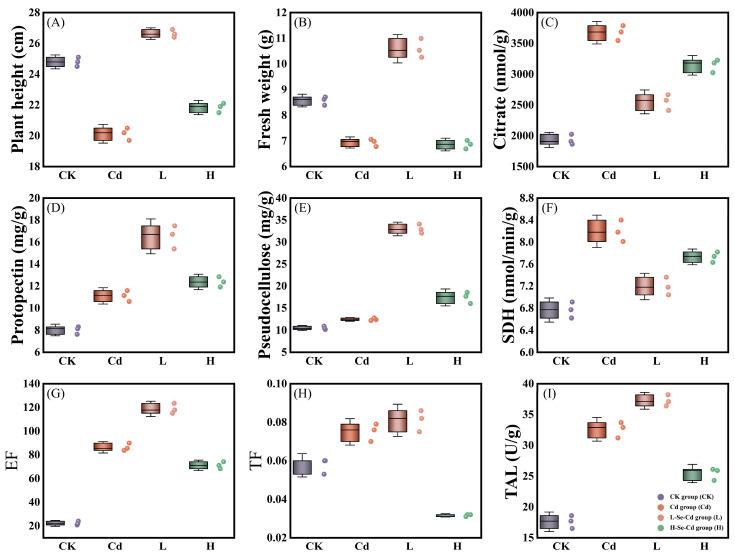
Physiological and biochemical indices of Cd response in rice roots under Se stress. (**A**,**B**) Plant height and fresh weight of rice in four treatments were determined. (**C**–**G**) Determination of citrate, protopectin, hemicellulose, SDH, and TAL in rice root system in four treatments. (**H**,**I**) Calculation of the contents of Cd enrichment factors and transporters in rice root systems in four treatments.

In the present study, rice seedlings were divided into four treatments, in which fresh weight and plant height showed the same trend. Compared with the control, fresh weight and plant height were significantly suppressed after adding Cd. After the addition of a low concentration of Se, not only did the inhibition phenomenon disappear, but it also promoted the growth of rice seedlings to some extent. Inhibition again occurs when the concentration of added Se is too high (Figure 2 and Figure S1). Afterward, protopectin, hemicellulose, and various enzymes in rice seedlings were determined. The experimental results showed that the concentration of protopectin in the Cd group and the group treated with a low concentration of Se showed a gradual increase. The hemicellulose concentration in the Cd group was only slightly higher than that in the control group, while the hemicellulose concentration increased significantly after the addition of a low concentration of Se. In contrast to low concentrations of Se, high concentrations inhibit the formation of protopectin and hemicellulose (Figure 2D,E). The changes in citrate and SDH showed the same trend. Cd significantly promoted the synthesis of both substances compared to the control group and was then inhibited after adding low concentrations of Se. At the same time, the addition of high concentrations of Se boosted it in contrast to low concentrations of Se, but the boosting effect was not as pronounced as that of the Cd group (Figure 2C,F). Changes in TAL were again different, with the enzyme synthesis progressively promoted in the Cd group and the low-concentration Se treatment compared to the control group. However, the promotion was reduced in the high-concentration Se treatment (Figure 2G). The EF for Cd was similar to the changes in TAL (Figure 2H). The changes in Cd’s TF were again different, showing a gradual increase in the TF in the Cd group and the low-concentration Se treatment compared to the control group. In contrast, the transporter factor of Cd was inhibited in the high-concentration Se treatment (Figure 2I).

### 3.2. Proteomic Analysis of Rice Cell Wall Polysaccharides

**Figure 3:**
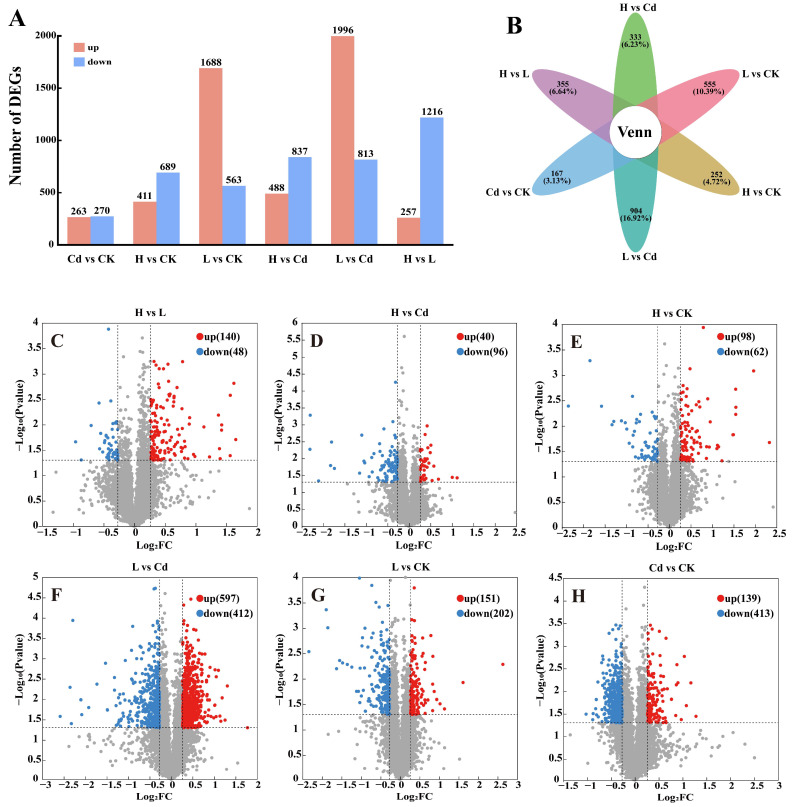
Differently expressed genes and proteins in six contrasting transcriptomes and proteomes. (**A**) Number of up- and down-regulated genes in rice roots in six contrasts. (**B**) Venn diagrams of unique genes shared by the six contrasts. (**C**–**H**) Number of up- and down-regulated proteins in rice roots in six contrasts.

We compared differentially regulated proteins for the four treatment groups (Figure 3C–H). A total of 136 differentially regulated proteins (e.g., OsRLCK89 and OsVPS2, etc.) were identified in H vs. Cd, and 96 were down-regulated and 40 were up-regulated (Figure 3D). In L vs. Cd by 1009 differentially regulated proteins (e.g., PsbR, OsMT3a, PsaK, and PsaN, etc.), 412 were down-regulated, and 597 were up-regulated (Figure 3F). There were 552 differentially expressed proteins (e.g., OsSGT, RBBI3, and OsPR1, etc.), 413 of which were down-regulated and 139 of which were up-regulated in Cd vs. CK (Figure 3H).

**Figure 4:**
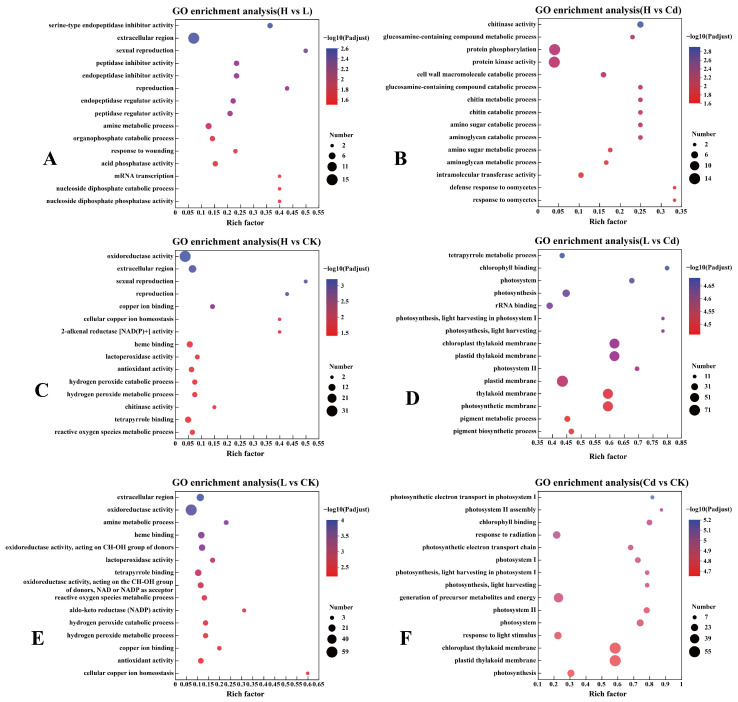
GO enrichment analysis of differentially expressed proteins in the rice root proteome in six contrasting groups. (**A**) H vs. L, (**B**) H vs. Cd, (**C**) H vs. CK, (**D**) L vs. Cd, (**E**) L vs. CK, and (**F**) Cd vs. CK.

The six contrasting differentially regulated proteins were subjected to GO and KEGG enrichment analyses for the top 15 enriched pathways expressed. The GO enrichment analysis showed that the differentially regulated proteins in H vs. Cd were mainly enriched in “protein kinase activity“ and “protein phosphorylation“. The differentially regulated proteins in L vs. Cd were mainly enriched in “plastid thylakoid membrane”, “chloroplast thylakoid membrane”, “plastid membrane”, “thylakoid membrane”, and “photosynthetic membrane“. Whereas in Cd vs. CK, differentially regulated proteins were mainly enriched in “precursor metabolites and energy production”, “chloroplast membranes”, and “plastid vesicle membranes” (Figure 4).

The KEGG enrichment analysis showed that “biosynthesis of secondary metabolites” and “metabolic pathways” were the main pathways enriched for differentially regulated proteins when comparing the two groups, L versus Cd and Cd versus CK. In contrast, the differentially regulated proteins in H versus Cd were mainly enriched in “Metabolic pathways” (Figure S2). At the same time, we also identified three protein pathways related to the cell wall, namely “Glycosyl hydrolases family 17”, “O-methyltransferase”, and “Polygalacturonase”, with 29, 29, and 16 proteins, respectively (Table S2).

### 3.3. Transcriptome Analysis of Rice Cell Wall Polysaccharides

The four subgroups were compared two by two, and three were selected for follow-up: H vs. Cd, L vs. Cd, and Cd vs. CK. A total of 1325, 2809, and 533 differentially expressed genes were identified by transcriptome analysis. There were 488 up-regulated genes (e.g., OspPLAIIdelta, CCR1, and TPS3, etc.) and 837 down-regulated genes in H vs. Cd, 1996 up-regulated genes (e.g., OspPLAIIdelta, OsLAX5, OsGSTU5, and U2, etc.) and 813 down-regulated genes in L vs. Cd, as well as 263 in Cd vs. CK up-regulated genes (e.g., OsSTA138 and OsRLCK332, etc.) and 270 down-regulated genes (Figure 3A). Venn analysis of differentially expressed genes did not find genes that could be present simultaneously in all six comparison groups at the same time (Figure 3B).

To further understand the function of differentially expressed genes, we mapped the four differentially expressed genes grouped for comparison to the GO database and functionally annotated them separately (Figure S3). GO functional annotation results showed that single genes in six comparisons were categorized into molecular functions, cellular components, and biological processes. The corrected *p*-value < 0.05 was considered significantly enriched for the GO term. In molecular function, almost all of the differentially expressed genes obtained from the six comparator groups mapped to “binding” (GO:0005488) and “catalytic activity” (GO:0003824). In terms of cellular components, most of the differentially expressed genes were labeled in the comparison as “membrane” (GO:0016020), “cellular fraction” (GO:0044464), “membrane fraction” (GO:0044425), and “organelle “(GO:0043226). Among the biological processes, the highest enrichment of differentially expressed genes was found in “metabolic processes” (GO:0008152) and “cellular processes” (GO:0009987).

**Figure 5:**
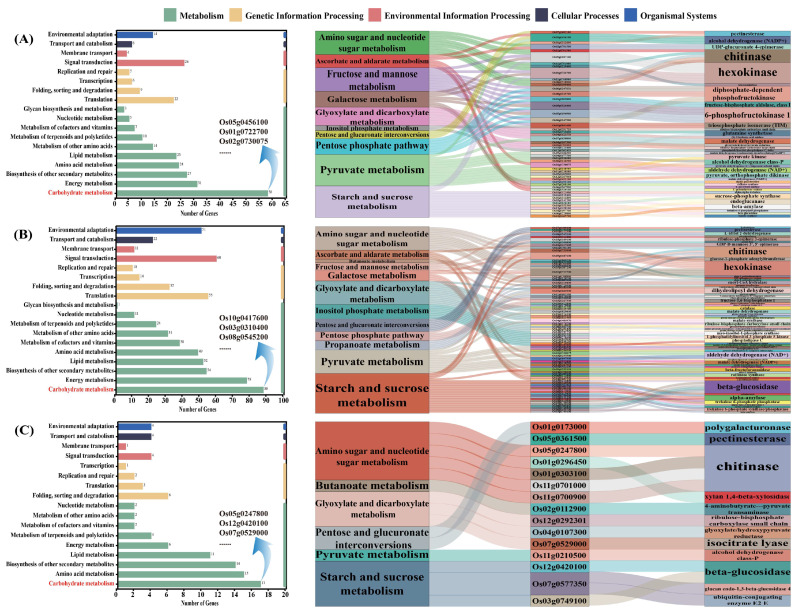
Functional annotation of differentially expressed genes in the rice root transcriptome. KEGG terms:(**A**) H vs. Cd, (**B**) L vs. Cd, (**C**) Cd vs. CK. Assignment of graphene oxide terms for different categories of metabolism, genetic information processing, environmental information processing, cellular processes, and organismal systems, and the pathways of carbohydrate metabolism corresponding to genes and enzymes.

**Figure 6:**
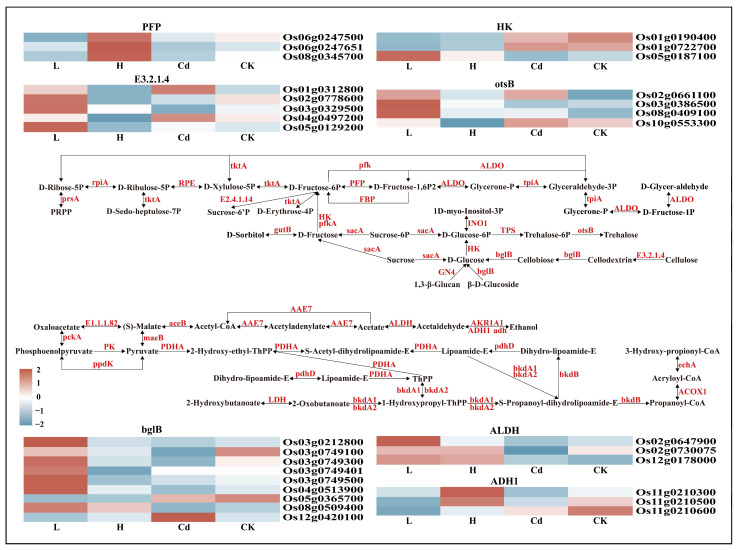
The proposed pathway for rice polysaccharide biosynthesis. The expression levels of individual genes encoding enzymes at each step are shown. The four columns correspond to L, H, Cd, and CK comparisons. Colors indicate log2-fold change in values (stressed vs. control).

To explore the most important biological processes in the rice root system, this study localized the differentially expressed genes in six comparative groups to the KEGG database, with 93 single genes localized to 18 pathways in H vs. Cd, 107 single genes localized to 18 pathways in L vs. Cd, and 53 single genes localized to 17 pathways in Cd vs. CK. The most representative KEGG functional category is “Carbohydrate metabolism”, followed by “Energy metabolism”, “Biosynthesis of other secondary metabolites”, “Amino acid metabolism”, and “Lipid metabolism”. Among them, the most significant number of single genes were localized to the “carbohydrate metabolism” subcategory, which could be subdivided into 15 pathways, and the genes corresponding to each pathway and the enzymes corresponding to the genes were identified in this study (Figure 5 and Figure S4). The number of single genes involved in polysaccharide biosynthesis in H vs. Cd, L vs. Cd, and Cd vs. CK was 27, 34, and 4, respectively. Their corresponding enzymes included β-glucosidase, endoglucanase, alginate 6-phosphate phosphatase, hexokinase, diphosphate-dependent fructofuranose phosphatase, ethanol dehydrogenase class P, and aldehyde dehydrogenase. Analysis of the transcriptomic data of key enzymes of “carbohydrate metabolism” in six comparisons identified potential biosynthetic pathways for cell wall polysaccharide formation in rice (Figure 6 and Figure S3).

### 3.4. Joint Transcriptome and Proteome Analysis of Rice Cell Wall Polysaccharide

**Figure 7:**
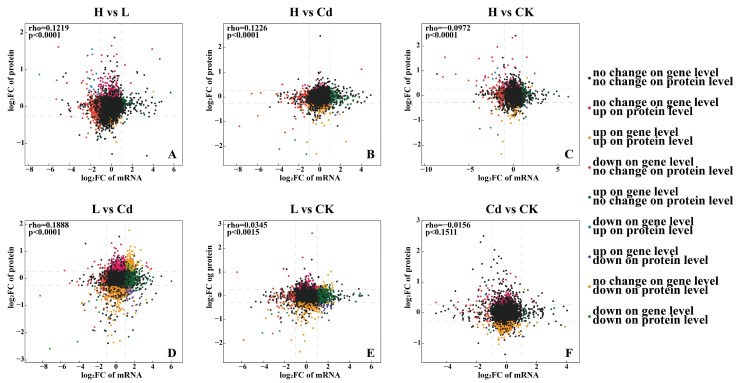
Correlation analysis of transcriptomics and proteomics data in rice roots under Se-mediated Cd stress. (**A**–**F**) Nine-quadrant plots represent cross-comparisons of genes and proteins. (**A**) H vs. L, (**B**) H vs. Cd, (**C**) H vs. CK, (**D**) L vs. Cd, (**E**) L vs. CK, and (**F**) Cd vs. CK. These values indicate the number of genes/proteins occurring in the different quadrants.

**Figure 8:**
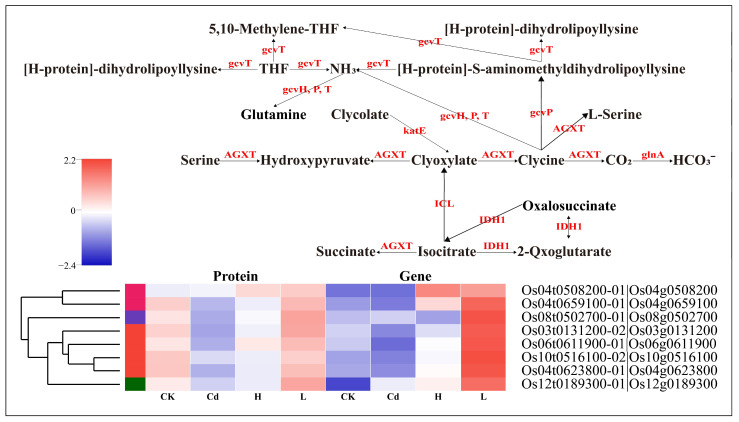
Se-mediated metabolic pathway analysis of transcriptomic and proteomic data in rice roots under Cd stress. The graphs show the expression levels of the shared genes/proteins encoding the enzymes at each step. The four columns correspond to CK, Cd, H, and L comparisons. Colors indicate a log2-fold change in values (stressed vs. control).

We correlated transcriptomic and proteomic data, and the results were most pronounced in L vs. Cd (Figure S5). To investigate the relationship between transcriptome responses and proteome responses, a nine-quadrant analysis was performed in this study on six comparison groups (Figure 7). Only five down-regulated genes/proteins in the H vs. Cd comparison showed similar trends in the transcriptome and proteome, 202 up-regulated and 12 down-regulated genes/proteins in the L vs. Cd comparison showed similar trends in the transcriptome and proteome, and one up-regulated and three down-regulated genes/proteins in the Cd vs. CK comparison showed similar trends. In addition, six genes/proteins showed opposite trends in H vs. Cd, 35 genes/proteins showed opposite trends in L vs. Cd, and four genes/proteins showed opposite trends in Cd vs. CK. In rice’s combined transcriptome and proteome analysis, 11 shared genes/proteins were also found in H vs. Cd, 253 shared genes/proteins in L vs. Cd, and 10 shared genes/proteins in Cd vs. CK (Table S4). On this basis, we found vital shared genes/proteins and corresponding enzymes and identified synthetic pathways (Figure 8). These shared genes/proteins were found to be up-regulated under Cd stress with the addition of low concentrations of Se, suggesting that low concentrations of Se significantly inhibited Cd activities.

## 4. Discussion

In this study, we analyzed the pathways in the cell walls in terms of transcriptome and proteome, found genes and proteins related to the cell wall, and finally identified genes/proteins common to both transcriptome and proteome. The metabolites of these genes/proteins are greatly useful in developing Cd-tolerant crop varieties. Previous studies have demonstrated the existence of antagonism between Se and Cd in plants [10]. In this study, the rice variety was Chunyou 987, a japonica rice, divided into four treatment groups. After 21 days of observation, it was found that the plant height and fresh weight of rice seedlings under Cd stress were significantly reduced, the same as the results of the previous study [28]. Plant height and fresh weight were significantly increased after the addition of low concentrations of Se, which indicated that the inhibitory effect of low concentrations of Se on Cd uptake was confirmed in previous studies. In contrast, plant height and fresh weight were again inhibited after adding high concentrations of Se (Figure 2 and Figure S1). There is a mutual inhibition between Se and Cd, which must have a critical point. Gao et al. demonstrated this in their previous study and found a threshold for mutual inhibition of Se and Cd, which corroborates the conjecture of the present study [29].

Cellulose and hemicellulose form a network with many different types of modified cell wall proteins [30]. It has been shown that analysis of Germin-like Protein Genes (OsGLPs) leads to meaningful changes in cellulose and protopectin content in blr1D leaves, while pectin methyl esterase and cellulose synthase lead to reduced pectin and cellulose content and cell wall abnormalities [31]. However, there are no studies on the effects of Cd and Se on protopectin and hemicellulose, so one can venture a guess based on the results of the present experiment that Cd and low concentrations of Se promote the synthesis of protopectin and hemicellulose, and that high concentrations of Se inhibit the formation of protopectin and hemicellulose compared to low concentrations of Se (Figure 2D,E). Matrix polysaccharides are the major contributors to the weight of the cell wall, where functional groups are also the primary bearers of metal cation binding by cell wall polysaccharides [32]. The argument that there are other important metal-binding sites in the cell walls of plants in the family Gramineae is widely accepted [33].

In rice, citrate, SDH, and TAL are closely related to the synthesis and metabolism of the cell wall. Citrate is a weak triprotonic acid with multiple ligands. There is a solid binding force with heavy metal ions, and the removal of Cd by citrate mainly involves two processes: acidolysis and chelation [34]. When the balance of anions and cations in the plant body is out of balance, it can be regulated by citrate, thus enhancing the resistance to heavy metal ions [30]. Citrate can also enhance the chelation of different heavy metals by increasing the solubility and mobility of the metals [35]. It has been shown that citrate in plants significantly reduces Cd [36]. After being contaminated with Cd, plants self-regulate and inhibit Cd uptake by increasing the citrate content in the body, which is the same as the results of this experiment (Figure 2C). SDH plays a crucial role in cell wall metabolism and catalyzes succinate’s oxidative dehydrogenation [37]. It has been shown that the iron-sulfur subunit of SDH (SDH2) plays a crucial role in electron transfer in plant mitochondria and affects rice leaf senescence and yield [38]. Succinate dehydrogenase inhibitors (SDHI) inhibit some bacteria in rice; for example, cyclobutrifluram has a strong inhibitory effect on *Vibrio* spp. [39]. The study demonstrated that SDHI was inhibited. SDH content increased in plants under Cd pollution stress, thus inhibiting Cd uptake (Figure 2F). TAL is an enzyme found mainly in grasses that increases the strength of cell walls by deaminating l-tyrosine to coumaric acid and has a significant inhibitory effect on stripe rust of barley [40]. TAL affects cell wall synthesis and structure primarily by catalyzing the oxidative deamination of tyrosine [41]. However, no studies on the effects of Cd and Se on TAL were found, and the results of the experiment showed that it can be hypothesized that Cd and low concentrations of Se promote TAL synthesis. In contrast, high concentrations of Se produce an inhibitory effect (Figure 2G).

Current research does not indicate a biological function for Cd. Cd can only enter plant cells by utilizing transporters for other divalent cations (Calcium, Zn, and Fe, among others) [42,43]. The inhibitory capacity of plants for heavy metals can be estimated by the magnitude of EF and TF [44]. Low concentrations of Se increase the metal transporters available on the roots, thus promoting Cd translocation and enrichment, whereas the opposite is true for high concentrations of Se (Figure 2H,I). In summary, cell walls contaminated with Cd secrete derivation-related substances to resist Cd stress.

The uptake and transportation of metals in plants is a highly complex process that requires the simultaneous action of several different transport proteins [45]. Large amounts of pectin, cellulose, and hemicellulose are present in the plant cell wall, and due to the hydroxyl, carboxyl, and sulfur groups in these substances, the cell wall can bind to Cd and prevent Cd from entering the plant [46]. In plant cell walls, polysaccharides play a vital role in metal binding and accumulation [47]. Differential regulation of transporter proteins involved in Cd uptake and transport in cell wall polysaccharides during exposure of rice seedlings to Cd, low concentrations of Se + Cd, and high concentrations of Se + Cd may be responsible for the reduced accumulation in rice. To find out which proteins play a role in the relationship between Se and Cd, GO and KEGG enrichment analyses of six contrasting differentially regulated proteins were performed in this study. The GO enrichment analysis showed that the differentially regulated proteins in H vs. Cd were mainly enriched in “protein kinase activity” and “protein phosphorylation”. In L vs. Cd, differentially regulated proteins were mainly enriched in “plastid thylakoid membrane”, “chloroplast thylakoid membrane”, “plastid membrane”, “thylakoid membrane”, and “photosynthetic membrane“ in Cd vs. CK. The differentially regulated proteins in Cd vs. CK were mainly enriched in “chloroplastic membrane”, “chloroplast thylakoid membrane”, “plastid thylakoid membrane”, and “generation of precursor metabolites and energy” (Figure 4). The proteins that resist Cd in plants mainly relate to the cell wall and cell membrane. Adding a low concentration of Se mainly inhibits the toxicity of Cd through the membrane-related proteins. In contrast, the high concentration of Se regulates the stress of Cd through the phosphorylation of proteins and the activity of protein kinase. The results of the KEGG enrichment analysis showed that in the comparisons of L and Cd and Cd and CK, the differentially regulated proteins were mainly enriched in “Metabolic pathways” and “Biosynthesis of secondary metabolites”, while in the comparison of H vs. Cd differentially regulated proteins were mainly enriched in “Metabolic pathways” (Figure S2). Carbohydrate metabolism is closely linked to plant cell walls [48]. This is in line with the transcriptome study (Figure 6). Three cell wall-related protein pathways were finally screened in this study as “Glycosyl hydrolases family 17”, “O-methyltransferase”, and “Polygalacturonase” with protein numbers of 29, 29, and 16, respectively (Table S2). A new study shows that “Glycosyl hydrolases family 17” is vital in stabilizing wheat yields [49]. Martin et al. found that “O-methyltransferase” is closely related to lignin in the cell wall and affects the efficiency of cell wall glycation [50]. Polygalacturonase plays a vital role in the degradation of demethylated homogalacturonan acid in the plant cell wall and is involved in the control of rice plant height through the regulation of plant cell wall biosynthesis and hormone metabolism [51,52].

There is still much confusion about the role of cell wall polysaccharides in resistance to toxic heavy metals, especially because the molecular and genetic mechanisms that promote polysaccharide synthesis are still unclear [53]. When rice cells are exposed to toxic substances, functional groups such as hydroxyl, carboxyl, and aldehyde groups in the root cell wall bind to the toxic ions, thus preventing the toxic substances from entering the plant [54]. In order to further characterize the genes for key enzymes of polysaccharide synthesis induced by the effects of Cd and Se, the root systems of rice seedlings were subsequently subjected to transcriptome analysis. It has also been shown that the rice cell wall responds differently to Cd detoxification-related pathways under Cd stress conditions, including “AsA-GSH Cycle”, “cell wall formation and remodeling”, and “amino acid and terpene metabolism” [55]. The results showed that DEGs localized to metabolic pathways were mainly enriched in the “amino and nucleotide sugar metabolism” and “starch and sucrose metabolism” pathways. It was particularly evident in the “energy metabolism”, “carbohydrate metabolism”, and “amino acid metabolism” pathways (Figure 5). It was further shown that the content of cell wall polysaccharides regarding heavy metal binding was significantly increased under Cd stress. In the process of analysis using KEGG, 39 critical enzymes related to polysaccharide biosynthesis were identified (Table S2). Seven key enzymes with the most genes were found, namely bglB, E3.2.1.4, otsB, PFP, HK, ALDH, and ADH1, and these seven key enzymes were subjected to heatmap analysis in the case of the four treatment groups (Figure 6), which also confirmed the existence of antagonism between Se and Cd in plants [10]. The expression level of these enzymes in rice cells largely determines the extent of cell wall polysaccharide accumulation under Cd and Se stress, and the single-gene characterization of these enzymes may provide a basis for subsequent studies on other essential enzymes involved in cell wall polysaccharide biosynthesis [18].

Joint multi-omics analysis allows a better representation of gene and protein associations [28]. This study performed a nine-quadrant analysis of the proteome in conjunction with the transcriptome to further reveal the mechanism of action of rice cell walls on Se and Cd (Figure 7). In a combined transcriptome-proteome analysis of rice, 11 shared genes/proteins in H vs. Cd, 253 shared genes/proteins in L vs. Cd, and 10 shared genes/proteins in Cd vs. CK were also identified (Table S4). It can be seen that in the plant cell wall, low concentrations of Se inhibit the uptake and transportation of Cd, while high concentrations of Se hardly inhibit Cd and even promote it. It also provides ideas for further research.

## 5. Conclusions

In this study, we found that under Cd stress, rice cell wall polysaccharides spontaneously produce substances that prevent Cd from entering the plant. Low concentrations of Se would inhibit the uptake and transportation of Cd, while high concentrations of Se would have little inhibitory effect on Cd and even promote it. Comprehensive proteomic and transcriptomic analyses of rice seedlings screened three cell wall-related protein pathways, namely “Glycosyl hydrolases family 17”, “O-methyltransferase”, and “Polygalacturonase”. Genes and proteins associated with the cell wall were discovered, leading to the identification of seven enzymes involved in the highest abundance of polysaccharide biosynthesis, including E3.2.1.4, bglB, otsB, HK, PFP, ADH1, and ALDH, and ultimately to the identification of the critical cell wall gene/protein, Os03g0170500, among others. Ultimately, the molecular regulatory network of the rice cell wall response to Cd toxicity under Cd stress, after adding low concentrations of Se and high concentrations of Se, including both gene and protein products, was subsequently proposed. The current multi-omics study of rice cell walls provides a new way to study abiotic stresses in crops.

## Data Availability

Data available in a publicly accessible repository. The data presented in this study are openly available in National Center for Biotechnology Information(NCBI) database at https://www.ncbi.nlm.nih.gov/, reference number PRJNA1174742.

## Supplementary Materials

The following supporting information can be downloaded at: https://www.mdpi.com/article/10.3390/toxics13080642/s1, Figure S1: Physiological response of rice roots to cadmium under selenium stress; Figure S2: KEGG enrichment analysis of differentially expressed proteins in the rice root proteome in six contrasting groups; Figure S3: Functional annotation of differentially expressed genes in the rice root transcriptome in six comparisons; Figure S4: Functional annotation of differentially expressed genes in the rice root transcriptome; Figure S5: Scatterplot of correlation between proteomic and metabolomic association data; Table S1: Hoagland nutrient solution ingredients; Table S2: Proteins associated with the cell wall; Table S3: Gene corresponding enzyme; Table S4: Shared genes proteins.
