# Supplementary material for: Integrated Proteomic and Transcriptomic Analysis Reveals the Mechanism of Selenium-Mediated Cell Wall Polysaccharide in Rice (Oryza sativa L.) Cadmium Detoxification
Source: Toxics. 2025 Jul 30;13(8):642. doi: 10.3390/toxics13080642 (PMC12390211; doi:10.3390/toxics13080642)

Fig. S1 Physiological response of rice roots to cadmium under selenium stress. (A) Rice root growth in four treatments. (B) Rice stem growth in four treatments.

Fig. S2 KEGG enrichment analysis of differentially expressed proteins in the rice root proteome in six contrasting groups. (A) H vs L, (B) H vs Cd, (C) H vs CK, (D) L vs Cd, (E) L vs CK, and (F) Cd vs CK.

Fig. S3 Functional annotation of differentially expressed genes in the rice root transcriptome in six comparisons. GO terms for (A) H vs L, (B) H vs Cd, (C) H vs CK, (D) L vs Cd, (E) L vs CK, and (F) Cd vs CK. Assignment of graphene oxide terms for different categories of biological processes, molecular functions and cellular components.

Fig. S4 Functional annotation of differentially expressed genes in the rice root transcriptome. KEGG terms:(A) H vs. L, (B) H vs. CK, (C) L vs. CK. Assignment of graphene oxide terms for different categories of metabolism, genetic information processing, environmental information processing, cellular processes, and organismal systems.

Fig. S5 Scatterplot of correlation between proteomic and metabolomic association data. (A) H vs. Cd, (B) L vs. Cd, (C) Cd vs. CK.Colors indicate log2-fold change in values (stressed vs. control).

Fig. S1 Physiological response of rice roots to cadmium under selenium stress. (A) Rice root growth in four treatments. (B) Rice stem growth in four treatments.

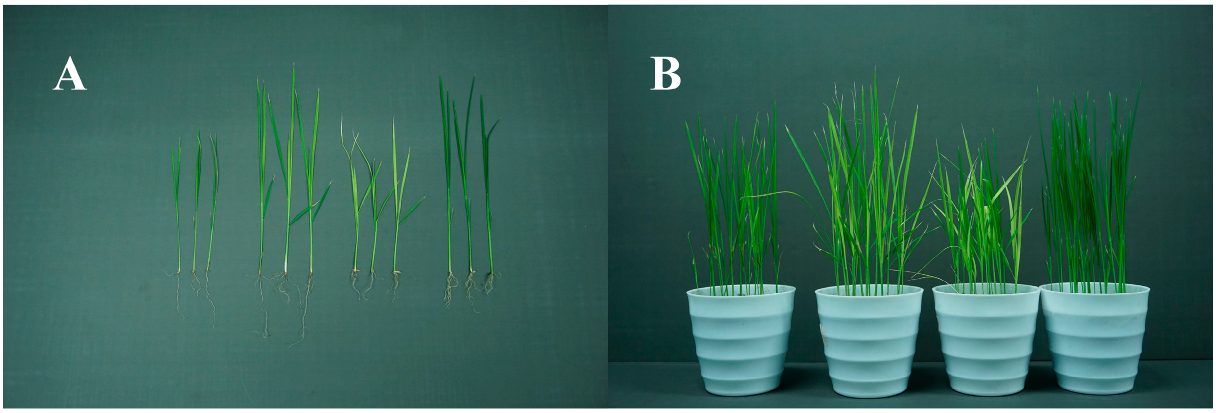

Fig. S2 KEGG enrichment analysis of differentially expressed proteins in the rice root proteome in six contrasting groups. (A) H vs L, (B) H vs Cd, (C) H vs CK, (D) L vs Cd, (E) L vs CK, and (F) Cd vs CK.

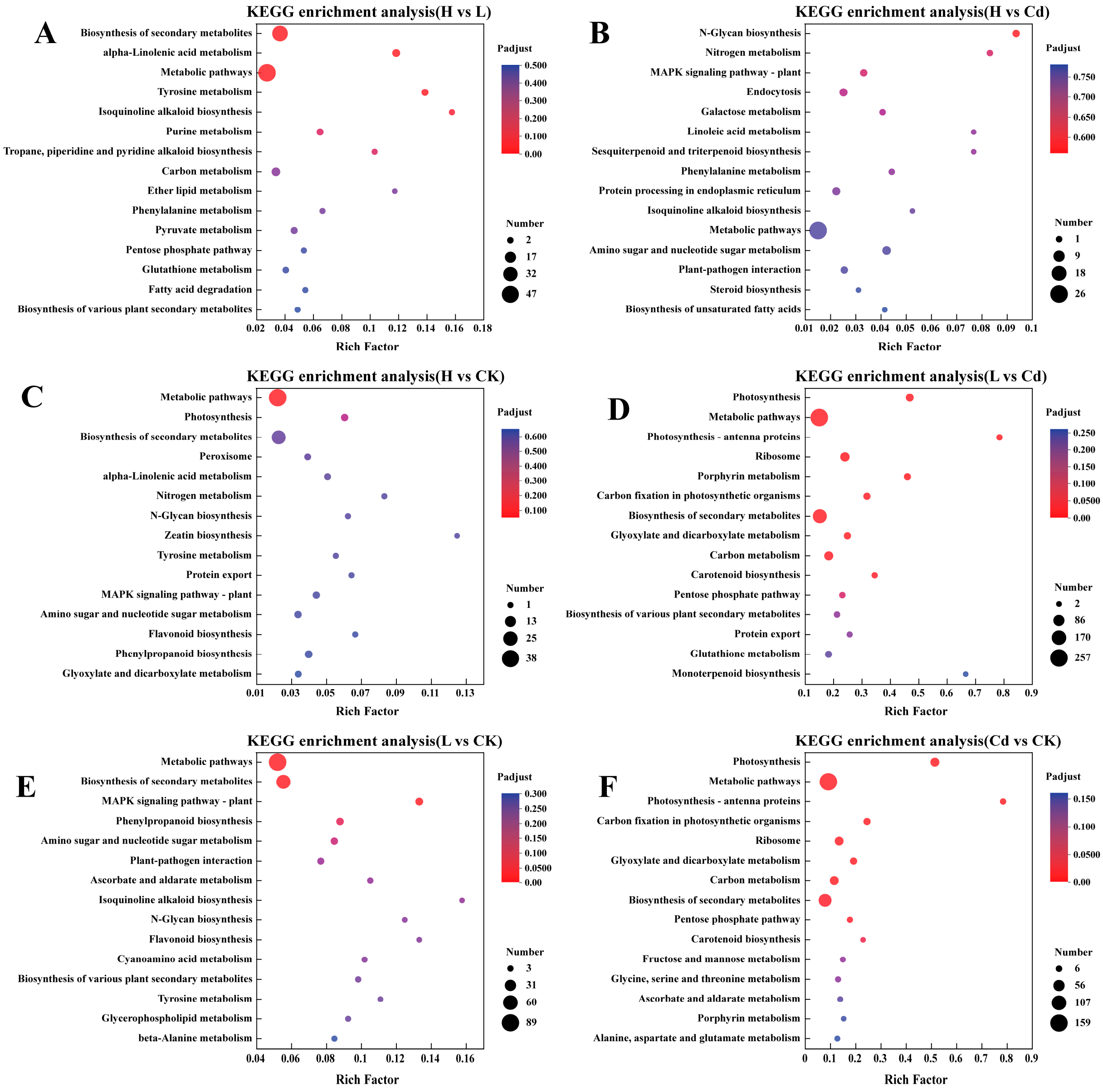

Fig. S3 Functional annotation of differentially expressed genes in the rice root transcriptome in six comparisons. GO terms for (A) H vs L, (B) H vs Cd, (C) H vs CK, (D) L vs Cd, (E) L vs CK, and (F) Cd vs CK. Assignment of graphene oxide terms for different categories of biological processes, molecular functions and cellular components.

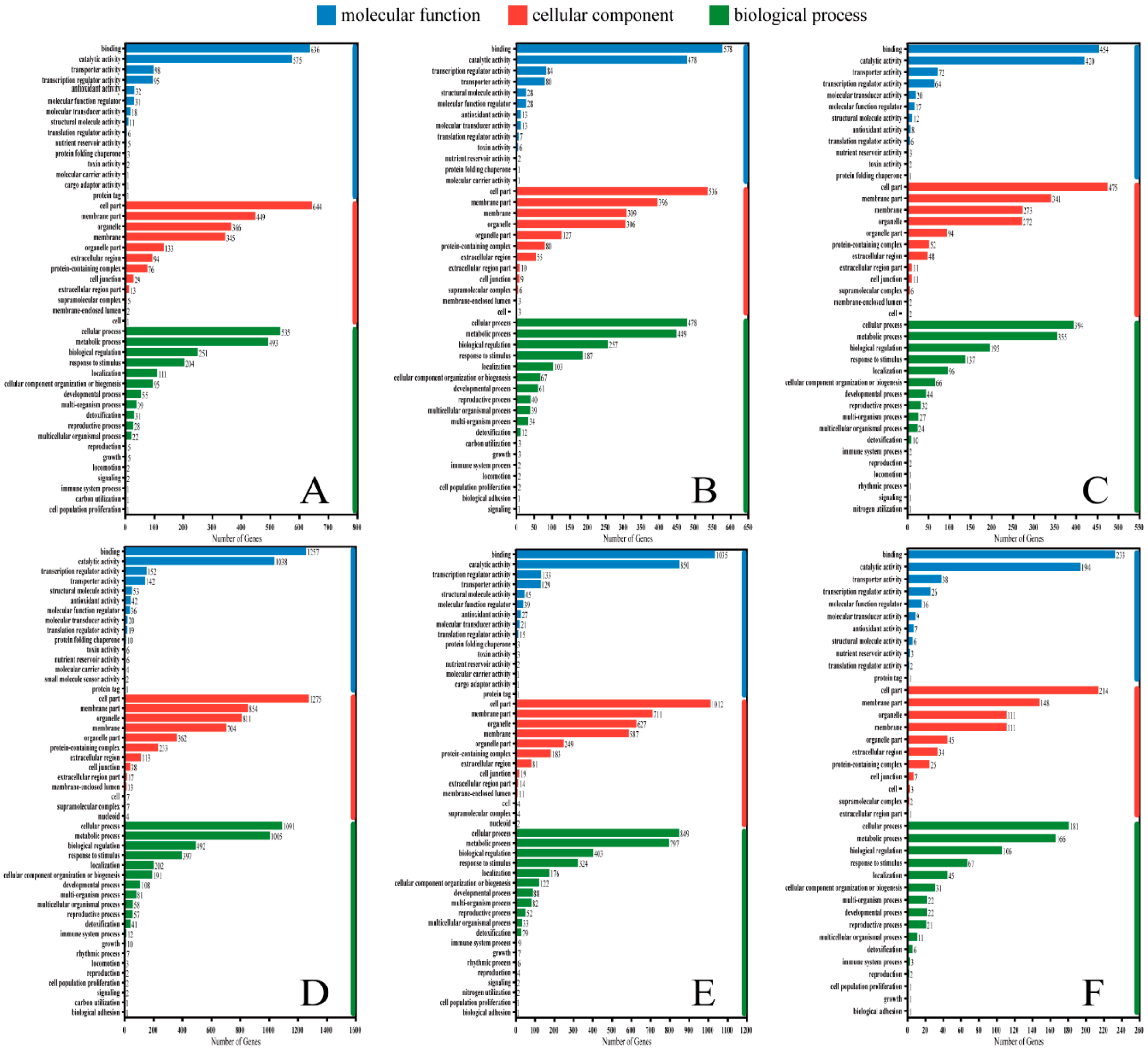

Fig. S4 Functional annotation of differentially expressed genes in the rice root transcriptome. KEGG terms:(A) H vs. L, (B) H vs. CK, (C) L vs. CK. Assignment of graphene oxide terms for different categories of metabolism, genetic information processing, environmental information processing, cellular processes, and organismal systems.

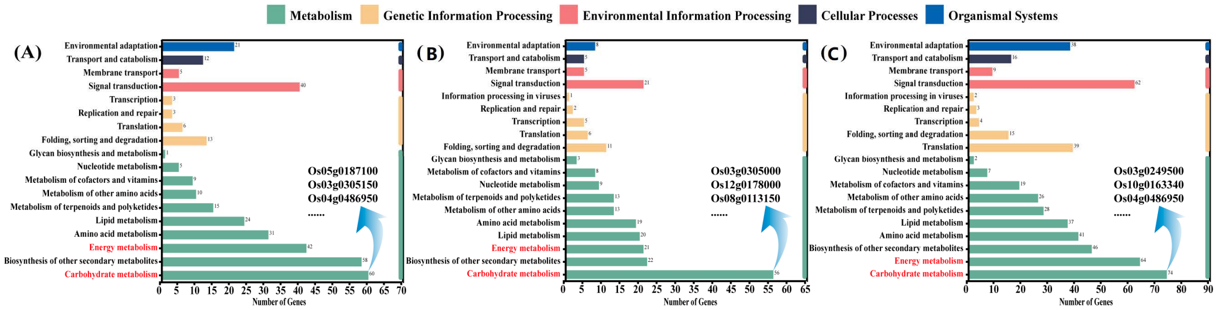

Fig. S5 Scatterplot of correlation between proteomic and metabolomic association data. (A) H vs. Cd, (B) L vs. Cd, (C) Cd vs. CK.Colors indicate log2-fold change in values (stressed vs. control).

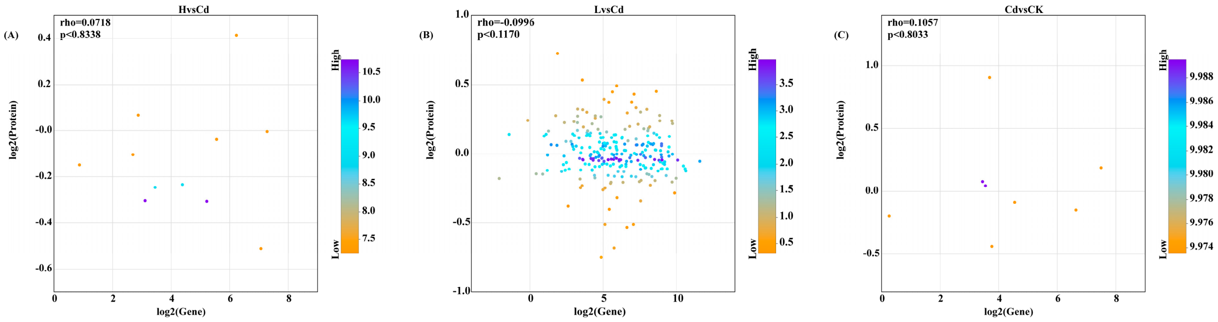

Supplement: Supplementary file 1 [file toxics-13-00642-s001.zip › toxics-3730897-supplementary.pdf]
